# Zero-valent iron/activated carbon microelectrolysis to activate peroxydisulfate for efficient degradation of chlortetracycline in aqueous solution

**DOI:** 10.1039/d0ra03639k

**Published:** 2020-05-20

**Authors:** Lu An, Pengfei Xiao

**Affiliations:** College of Forestry, Northeast Forestry University Harbin 150040 China xpfawd@nefu.edu.cn

## Abstract

Tetracycline antibiotics are widely used in human and veterinary medicine; however, their gradual increase in the aquatic environment poses a serious threat to human health and ecosystems. The reactivity of peroxydisulfate (PDS) in the degradation of chlortetracycline (CTC) in aqueous solution using a zero-valent iron/activated carbon (AC) microelectrolysis method (Fe^0^–AC/PDS) was investigated by batch experiments. The results showed that the effects of different systems were as follows: Fe^0^–AC/PDS > Fe^0^/PDS > AC/PDS > Fe^0^–AC > AC > Fe^0^ > PDS. In the Fe^0^–AC/PDS system, the degradation efficiency of CTC could reach 88% under the following optimal experimental conditions: Fe^0^ dose of 0.4 g L^−1^, PDS dose of 2 g L^−1^, pH of 3 and initial CTC concentration of 50 mg L^−1^. The presence of Cl^−^, HCO_3_^−^ and H_2_PO_4_^−^ inhibited the degradation of CTC, while humic acid accelerated the degradation rate of CTC. The mineralization of CTC was evaluated from the TOC, with a value of 31.44% in 7 h. Free radical identification experiments showed that SO_4_^−^˙ and O_2_^−^˙ were involved in the degradation of CTC. The iron and carbon materials had good reusability, and the degradation rate of CTC was still approximately 70% after four cycles. Finally, the possible mechanism for the degradation of CTC by the Fe^0^–AC/PDS systems was discussed. Based on the above conclusions, Fe^0^–AC microelectrolysis is a new heterogeneous catalytic method for green and efficient activation of PDS and demonstrates potential applicability in the treatment of wastewater.

## Abstract 2

The microelectrolysis system composed of zero-valent iron and activated carbon can effectively activate persulfate to produce SO_4_^−^˙ and O_2_^−^˙, which have excellent capacity for degradation of chlortetracycline hydrochloride.

## Graphical abstract

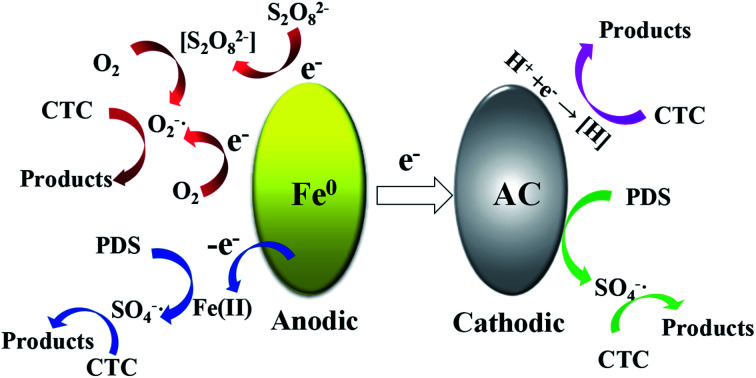

## 1. Introduction

Tetracyclines (TCs) are broad-spectrum antibiotics.^1^ Because of their advantages of high quality and low price,^2^ TCs have been widely used to improve human health, treat and prevent animal infection, and promote growth in animal husbandry.^3^ However, due to the essential characteristics of antibiotics, people and animals can only absorb and metabolize some of the TCs, and a considerable percentage (70%–90%) is released into the environment through wastewater effluent and animal manure.^4^ In addition, the excessive use of TCs will also result in them entering the environment through municipal effluent, sewage sludge, solid wastes and manure applications,^5^ causing significant toxicity and serious contamination. Therefore, it is crucial to study and promote the technology of removing TCs for maintaining a healthy environment.

Recently, various techniques have been applied to remove TCs from water, including adsorption, membrane separation, microbial degradation and advanced oxidation.^6–10^ Among the above methods, the advanced oxidation process is considered to be the most attractive and potential method to remove TCs, among which hydroxyl radicals (·OH) are one of the most important oxidants (*E*^0^ = 2.7 V).^11^ Although ·OH can oxidize and degrade many organic pollutants, its application is limited by factors such as its short life span and the pH (Fenton reaction).^12^ Compared with ·OH, SO_4_^−^˙ possesses a higher redox potential (*E*^0^ = 2.5–3.1 V), a longer lifetime (3–4 × 10^−5^ s) and a higher oxidation selectivity,^13^ so it is proposed as an alternative to ·OH. In general, SO_4_^−^˙ can be generated by the activation of persulfate (PS).

PS can be activated by heat, UV irradiation, ultrasound, bases, activated carbon (AC), phenols, and transition metal ions (Co^2+^, Fe^2+^).^14–20^ Iron species are widely used in wastewater treatment due to their low cost, nontoxicity and effectiveness. Although Fe^2+^ can rapidly activate PS to degrade organic pollutants,^21^ Fe^2+^ will be rapidly oxidized to Fe^3+^, resulting in the poor utilization of iron and PS during the experiment.^22^ In addition, excessive Fe^2+^ consumes the sulfate radical generated in the system, as shown in eqn (1):^23^

1 SO_4_^−^˙ + Fe^2+^ → Fe^3+^ + SO_4_^2−^

Therefore, many composite catalysts have been widely investigated, especially iron and its composites. Due to requiring no electricity, and its characteristics of high efficiency and low cost, iron–carbon microelectrolysis has been extensively used in wastewater treatment around the world. Han *et al.* confirmed that the iron–carbon microelectrolysis filler has good pretreatment performance for dye wastewater after introducing into circulation flow.^24^ In an iron–carbon microelectrolysis system, iron and activated carbon act as electrode materials, which form a large number of microbatteries and then form Fe(ii) and [H] in the reaction process.^25^ They are highly active and can decompose most organic pollutants.^22^ The reaction equation is as follows (eqn (2)–(4)):^26^

2 Anodic oxidation: Fe − 2e^−^ → Fe^2+^

3 Cathodic reduction: O_2_ + 4H^+^ + 4e^−^ → 2O˙ + 4[H] → 2H_2_O

In addition, coagulation, retention and adsorption reactions can also remove organic pollutants.^27^ However, the above processes easily harden, passivate and block after a period of operation in practical applications,^24^ so organic pollutants cannot be completely degraded. Therefore, iron–carbon microelectrolysis technology requires continuous improvement (such as the combination with PS) to become a time-efficient, versatile and adaptive alternative for the treatment of organic wastewater. Li *et al.* confirmed that the decolorization effect of iron–carbon microelectrolysis is activated PS on methyl orange was better than that of iron–carbon microelectrolysis alone.^27^ However, there is no study on the use of iron–carbon microelectrolysis as a PS activator to degrade TC.

In this study, a novel type of a zero-valent iron (Fe^0^)–activated carbon (AC) microelectrolysis method activated peroxydisulfate system (Fe^0^–AC/PDS) was investigated. A series of experiments were carried out with chlortetracycline (CTC) as the target pollutant. The objectives of this study are thus to (1) compare the degradation effect of Fe^0^–AC microelectrolysis/PDS with other systems and evaluate the effects of process parameters such as the Fe^0^/AC mass ratio and environmental conditions on degradation efficiency; (2) explore the mineralization degree of pollutants by measuring the total organic carbon (TOC) concentration; (3) elucidate the recovery of the Fe^0^–AC and the feasibility of multiple recycling steps; and (4) disclose the contribution of active oxygen species to the whole reaction system.

## 2. Experimental

### 2.1 Materials and chemicals

Sodium persulfate (Na_2_S_2_O_8_, 99% purity), chlortetracycline hydrochloride (CTC) (≥98% purity), ethanol (EtOH, 99%), *tert*-butyl alcohol (*T*BA, 99%), *para* quinone (*P*BQ, 99%), humic acid (HA, fulvic acid > 90%) and AC (with a diameter of 2 mm and specific surface area of 935 m^2^ g^−1^) were purchased from Aladdin Industrial Corporation. Zero-valent iron (99% purity), sodium chloride (NaCl, 99%), sodium bicarbonate (NaHCO_3_, 99%) and sodium dihydrogen phosphate (KH_2_PO_4_, 99%) were analytical grade and purchased from Sinopharm Chemical Reagent Corporation. Methanol, acetone and hexane were all high-performance liquid chromatography (HPLC) grade and supplied by Tianjin Kemiou Chemical Reagent Corporation. NaOH (0.5 M) and H_2_SO_4_ (0.5 M) solutions were utilized to adjust the pH value of the reaction solutions. Ultra-pure water prepared from a Millipore system (Pincheng, China) was used to prepare solutions for the experiments. Additionally, 100 mg L^−1^ CTC and 4 g L^−1^ PDS stock solutions were freshly prepared by dissolving CTC and PDS in ultra-pure water and then fully covering them with aluminum foil to avoid light. Prior to use, the activated carbon (AC, Sinopharm) was crushed to 120 mesh, and washed three times with ultrapure water and then oven-dried for 12 h at 80 °C.

### 2.2 Experimental device design

**Fig. 1:**
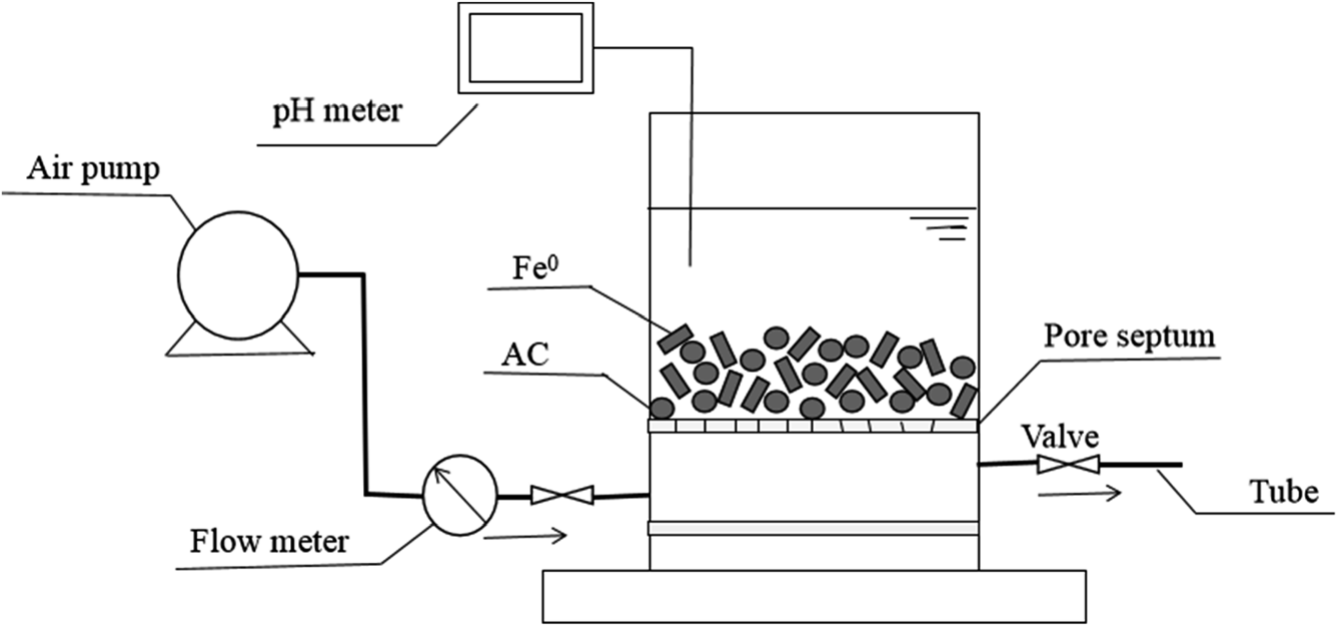
The schematic diagram of the reactor for continuous experiment.

For investigating the potential of Fe^0^–AC/PDS system in real application, the reactor for continuous experiment was constructed with air pump (ACO-003), rubber tube, air flow meter (LZB-3WB), pore septum, pH meter and a plexiglas cylinder (*ϕ* 6 cm × 8 cm, China) and the schematic diagram is shown in Fig. 1. The columnar reactor was filled with Fe^0^ and activated carbon.

### 2.3 Experimental procedures

Seven groups of experiments, PDS, AC, Fe^0^, Fe^0^–AC, AC/PDS, Fe^0^/PDS and Fe^0^–AC/PDS, were carried out with a sequencing batch operation mode in a plexiglas cylinder. The dose of Fe^0^ and AC were 0.4 and 0.2 g L^−1^, respectively. The other operating parameters were initial pollutant concentration of 50 mg L^−1^, PDS concentration of 2 g L^−1^ and pH values of 5.0. Various influencing factors were analyzed, including Fe^0^ doses of 0.067, 0.1, 0.2, 0.4 and 0.6 g L^−1^ (Fe^0^/AC mass ratio = 1 : 3, 1 : 2, 1 : 1, 2 : 1 and 3 : 1), PDS doses of 0.2, 0.5, 1, 2 and 3 g L^−1^, pH values of 3, 5, 7, 9 and 11, and initial pollutant concentrations of 20, 40, 60, 100 and 150 mg L^−1^. The concentrations of various inorganic anions (Cl^−^, HCO_3_^−^ and H_2_PO_4_^−^) were 100 mM, the doses of humic acid were 10, 20 and 50 mg L^−1^, and the concentrations of EtOH, *T*BA and *P*BQ were 50 mM. All samples were shaken well and then oscillated at room temperature (150 rpm) for regular sampling and analysis.

### 2.4 Analytical methods

After removing the sample, the solution was filtered using 0.45 µm filters. The concentration of CTC in the solution was determined by HPLC (Agilent 1260, USA). The HPLC analytical conditions were as follows: C18 column (pH range 8, 4.6 mm × 250 mm, 5 µm); mobile phase of 0.05 M ammonium oxalate solution–dimethylformamide–0.1 M diammonium hydrogen phosphate solution (56 : 40 : 4) (adjusted to a pH of 8.3 with an ammonia test solution); flow rate of 0.9 mL min^−1^; column temperature of 35 °C; detection at 370 nm. The injection volume was 20 µL, and the retention time of CTC was 3.8 min. The TOC concentration in solution was determined by a TOC analyzer (Shimadzu, Japan). The CTC concentration was analyzed by a first-order kinetic model:ln(*C*/*C*_0_) = −*k* × *t*where *t* is the reaction time, *C*_0_ and *C* are the initial CTC concentration and CTC concentration at time *t*, and *k* is the first-order kinetic reaction constant.

## 3. Results and discussion

### 3.1 Removal of CTC in different systems

**Fig. 2:**
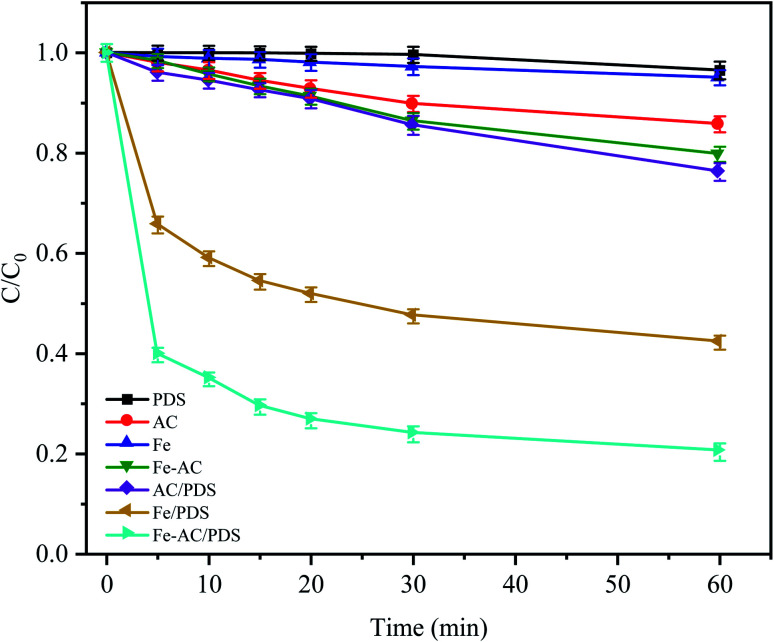
Degradation of CTC in different systems. *C*_0_ is the initial concentration of CTC and *C* is the concentration at time *t* (min). Experimental conditions: [CTC] = 50 mg L^−1^, [PDS] = 2 g L^−1^, [Fe^0^] = 0.4 g L^−1^, [AC] = 0.2 g L^−1^ and pH = 5.

The CTC removal efficiency of different combinations was compared (Fig. 2). In the Fe^0^ and AC treatment alone, only 4.96% and 14.26% of CTC were removed after 60 min, respectively, indicating that the adsorption capacity of AC and Fe^0^ to CTC is weak. In the Fe^0^–AC system, the removal rate of CTC was 20.24%, which showed that the oxidation ability of the active groups produced by Fe^0^–AC microelectrolysis was limited. At the same time, it was found that a small amount of CTC (approximately 3.51%) could be removed only by PDS after 60 min of reaction, indicating that PDS could not generate radicals by itself without a catalyst; thus, CTC could hardly be oxidized by PDS alone.^22^ In the AC/PDS system, the removal effect of CTC was not obvious (approximately 23.75%). The activated carbon could activate PDS, but the low dose of activated carbon was unable to sufficiently activate PDS to generate radicals.^18,28,29^

With the utilization of the Fe^0^/PDS and Fe^0^–AC/PDS systems, the reaction rates of the two treatments increased rapidly in the initial stage of reaction. In the Fe^0^/PDS system, 35% of CTC was removed In the first 5 min, indicating that iron could activate PDS to generate radicals, thus achieving the removal of CTC. Surprisingly, the reaction was further accelerated in the Fe^0^–AC/PDS system, and the removal rate of CTC reached 60.26% within 5 min of reaction. This demonstrated that the removal rate of the Fe^0^–AC/PDS system was faster than that of the Fe^0^/PDS system during the whole reaction period. Therefore, the primary battery system formed by Fe and AC aided the activation of PDS for the removal of CTC and accelerated the degradation efficiency. This phenomenon might be due to the presence of an AC-promoted electron transfer in the system, accelerating the formation of Fe^2+^ to generate SO_4_^−^˙.^30^ Furthermore, some studies have shown that current can promote the activation of persulfate. The results showed that the Fe^0^–AC microelectrolysis system could generate more oxidation radicals by activating PDS, which was more conducive to the removal of CTC.

### 3.2 Effect of the Fe^0^ dose on CTC degradation

**Fig. 3:**
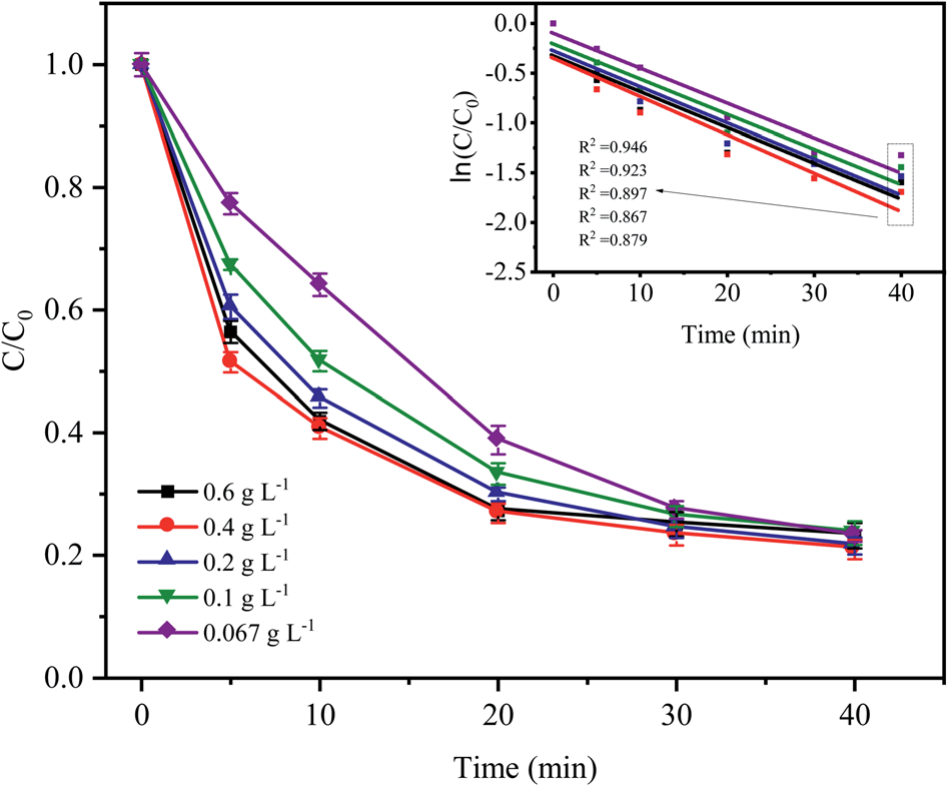
Effect of Fe^0^ dose on the degradation of CTC in Fe^0^–AC/PDS system. Experimental conditions: [CTC] = 50 mg L^−1^, [PDS] = 2 g L^−1^, [AC] = 0.2 g L^−1^ and pH = 5.

Fig. 3 shows the variations in CTC degradation efficiency by PDS activated by microelectrolysis at different Fe^0^ doses. When the Fe^0^ dose was 0.4 g L^−1^, the apparent rate constant and degradation rate reached the maximum with values of 0.0386 min^−1^ and 82.17% (after 60 min), respectively. With an increase in the Fe^0^ dose, more iron ions leached out in the early stage of the reaction, which promoted the formation of radicals and the degradation efficiency of CTC. However, when the Fe^0^ dose reached 0.6 g L^−1^, the degradation efficiency of CTC decreased. According to the principle of electrolytic reactions, Fe^0^ the anode loses electrons to form Fe^2+^ in the solution. When the Fe^0^ dose was relatively small (the amount of iron powder was less at this time), the contact area of Fe^0^ and AC was reduced, and the reaction of the primary battery was not sufficient, resulting in less SO_4_^−^˙ generated by the activated PDS. With an increase in Fe^0^ dose, the amount of iron involved in the reaction accumulated, which accelerated the reaction rate of the primary battery, resulting in an increase in SO_4_^−^˙. However, when the Fe^0^ dose was too high, the excessive iron powder in the system may react with H^+^ in the primary battery, which is not conducive to the electrochemical reaction. Additionally, as the reaction proceeded, the excess iron ions competed and consumed SO_4_^−^˙,^31^ leading to a decrease in the CTC degradation efficiency. The results suggested that a high or low Fe^0^ dose could inhibit the degradation of CTC, and the selection of an appropriate Fe^0^ dose was important for the reaction, while reducing the cost of the actual wastewater treatment.

### 3.3 Effect of the PDS dose on CTC degradation

**Fig. 4:**
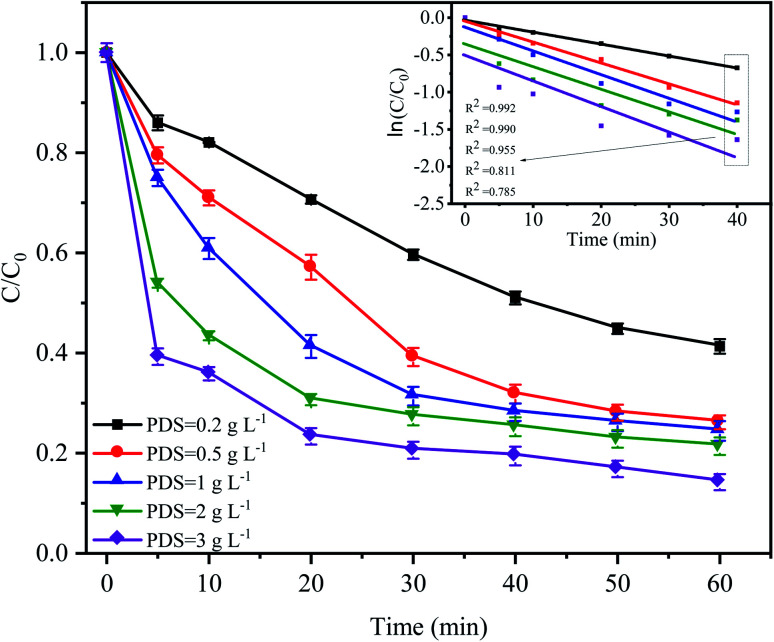
Effect of PDS dose on CTC degradation in Fe^0^–AC/PDS system. Experimental conditions: [CTC] = 50 mg L^−1^, [Fe^0^] = 0.4 g L^−1^, [AC] = 0.2 g L^−1^ and pH = 5.

The PDS concentration is a critical parameter for the activation of S_2_O_8_^2−^ in the advanced oxidation process since PDS is the source of the sulfate radicals. The effect of the PDS dose on CTC degradation in the system was investigated by varying the PDS concentrations from 0.2 to 3 g L^−1^. Fig. 4 shows that a significant difference in the CTC degradation rate was found between the treatments with different concentrations of PDS. Especially in the first 20 min of the reaction, the difference in degradation rate between adjacent treatments could reach approximately 10%. However, when the concentration of PDS continued to increase from 0.5 to 3.0 g L^−1^ after 60 min of reaction, the degradation rate of CTC increased relatively slowly; and the corresponding apparent rate constants were between 0.015 and 0.025 min^−1^. At low initial PDS concentrations, increasing the PDS dose would lead to an increase in SO_4_^−^˙, which would enhance the oxidative capacity of the entire system and ultimately improve the degradation rate of CTC.^32,33^ However, at high PDS concentrations, it would affect the degradation rate of CTC to a certain extent; SO_4_^−^˙ tended to be saturated because it could be converted into SO_4_^2−^ by a reaction with S_2_O_8_^2−^, which was accompanied by the formation of S_2_O_8_^−^˙ with insufficient oxidative capacity.^12,34^ These results suggested that an increase in oxidant content had a positive effect on pollutant degradation.

### 3.4 Effect of the initial pH on CTC degradation

**Fig. 5:**
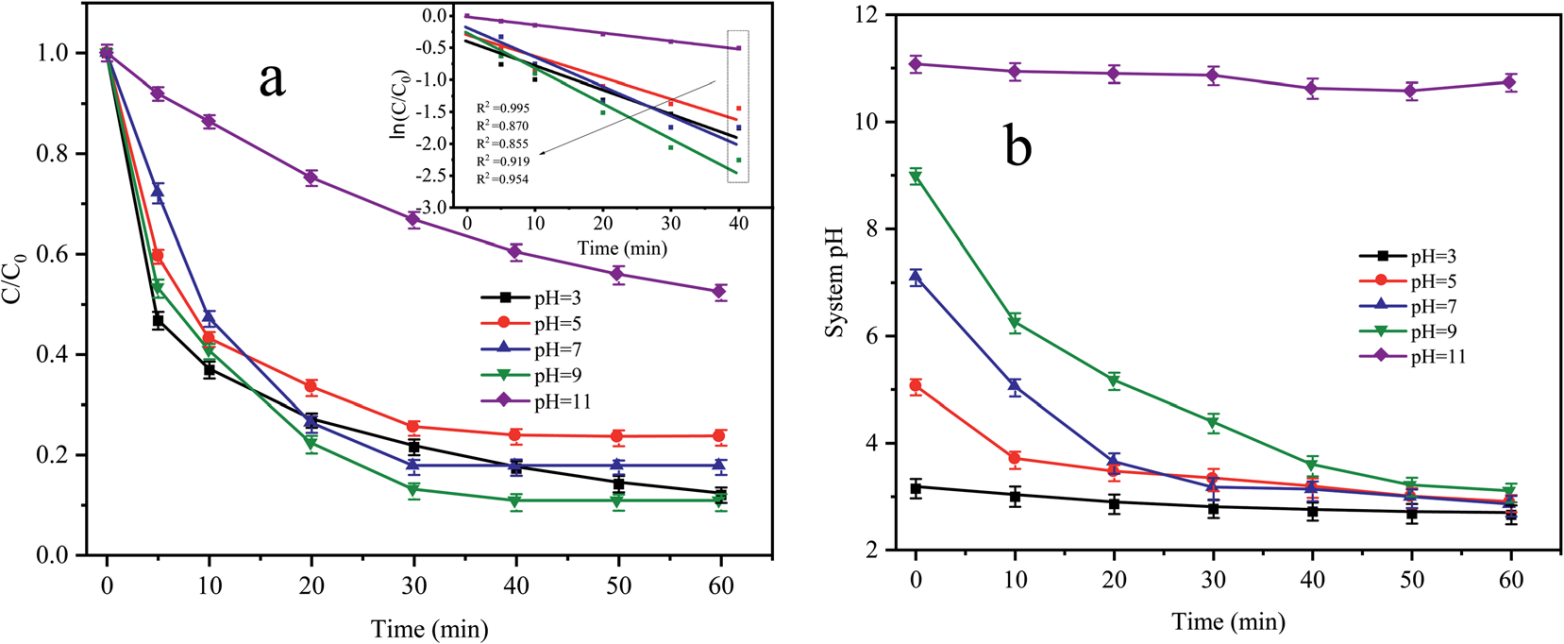
(a) Effect of initial pH on CTC degradation in Fe^0^–AC/PDS system and (b) variation curve of system pH during the reaction. Experimental conditions: [CTC] = 50 mg L^−1^, [PDS] = 2 g L^−1^, [Fe^0^] = 0.4 g L^−1^ and [AC] = 0.2 g L^−1^.

Fig. 5a shows the degradation effect and kinetic fitting effect of CTC under different pH conditions, and Fig. 5b shows the pH variation curve during the reaction. The removal of CTC in the Fe^0^–AC/PDS system followed the first-order kinetic model. When the initial pH values were 3, 5 and 7, the apparent rate constants were between 0.0214 and 0.03 min^−1^, and the corresponding CTC removal efficiency was 76.56% to 82.48% within 60 min of reaction. When the pH value increased to 9, the apparent rate constant reached the maximum with values of 0.0385 min^−1^. However, when the pH value reached 11, the apparent rate constant decreased to the lowest values of only 0.0109 min^−1^. When the initial pH was 3, the pH of the system changed little during the whole treatment. However, when the initial pH was in the range of 5 to 9, the pH of the system clearly decreased during the whole treatment process before they all finally decreased to approximately 3. In the treatment system with a pH of 11, the pH of the system was almost constant throughout the whole process. When the pH value of the solution was lower, more H^+^ accumulated in the iron–carbon primary battery and the reaction rate of the iron–carbon micromotor increased. At the same time, microelectrolysis accelerated the formation of Fe^2+^ and the electron transfer process, which promoted the activation of PDS and accelerated the degradation of CTC. However, the strong alkaline environment is not conducive to the formation of sulfate radicals by PDS activation; therefore, the degradation of CTC was inhibited.

### 3.5 Effect of the initial CTC concentration on CTC degradation

**Fig. 6:**
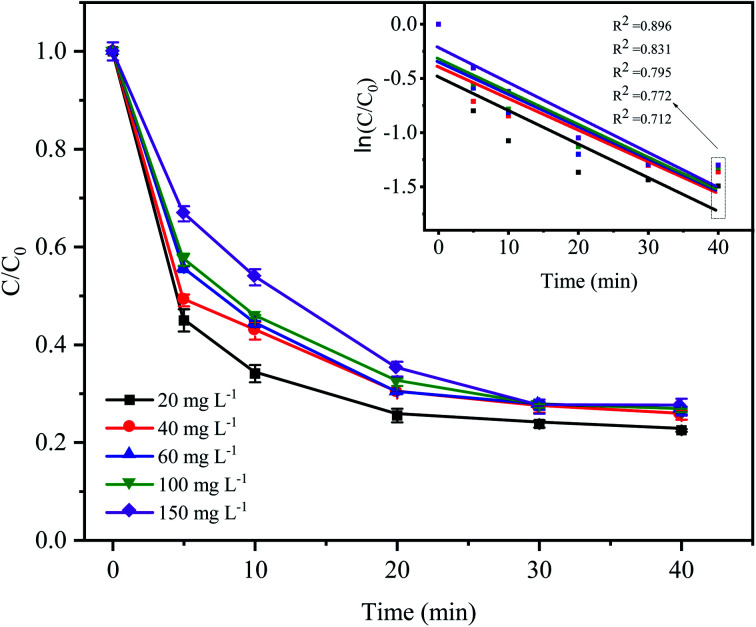
Effect of the initial CTC concentration on CTC degradation in Fe^0^–AC/PDS system. Experimental conditions: [PDS] = 2 g L^−1^, [Fe^0^] = 0.4 g L^−1^, [AC] = 0.2 g L^−1^, and pH = 5.

As shown in Fig. 6, the degradation rate of CTC decreased with increasing initial pollutant concentration. When the initial concentration of CTC increased from 20 mg L^−1^ to 150 mg L^−1^, the removal efficiency of CTC within 20 min of reaction was 74.49%, 69.84%, 69.9%, 67.64% and 64.96%, which showed a significant downward trend. The degradation efficiency of organic pollutants depended on the production of SO_4_^−^˙ and the reactions between the generated radicals and CTC. Because the concentration of PDS and the amount of catalyst in the system remained constant, the amount of generated free radicals would not change; therefore, the increase in initial pollutant concentration would decrease the probability of a reaction between the CTC molecules and reactive species.^35^ In addition, a great deal of byproducts and intermediates produced during the oxidation process might compete with the parent pollutants in the reaction with SO_4_^−^˙, which led to a reduction in removal efficiency.^36–38^ It is worth noting that when CTC concentration was 150 mg L^−1^, the apparent rate constant achieved the maximum of 0.0323 min^−1^. The results indicated that the Fe^0^–AC/PDS system could degrade CTC effectively even if CTC concentration was high, which showed that the method was suitable for a wide range of pollutant concentrations.

### 3.6 Effects of anions on CTC degradation

**Fig. 7:**
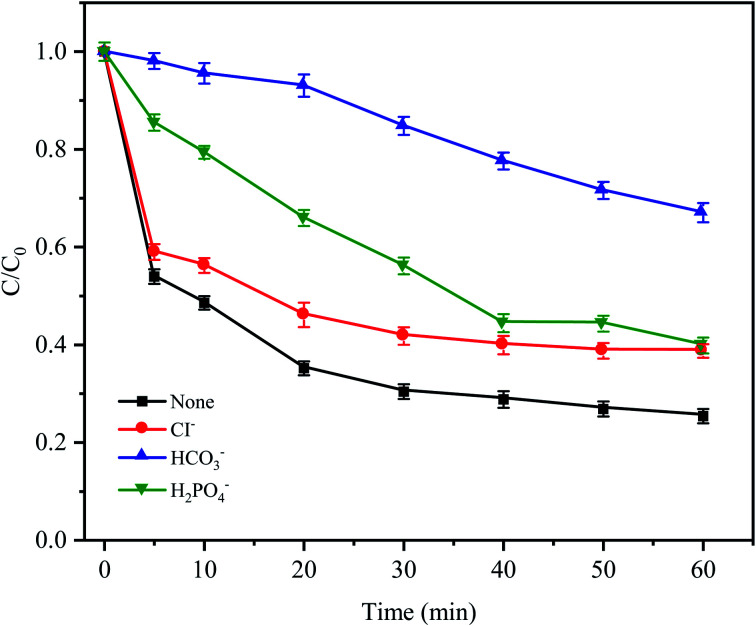
Effects of anions on CTC degradation in Fe^0^–AC/PDS system. Experimental conditions: [CTC] = 50 mg L^−1^, [PDS] = 2 g L^−1^, [Fe^0^] = 0.4 g L^−1^, [AC] = 0.2 g L^−1^, pH = 5 and [Cl^−^] = [HCO_3_^−^] = [H_2_PO_4_^−^] = 100 mM.

In natural water and wastewater systems, a large number of inorganic anions ubiquitously exist. There is no doubt that anions affect the oxidative degradation of antibiotics because some anions can react with free radicals. As depicted in Fig. 7, it was found that three typical inorganic anions had an inhibitory effect on CTC removal, and the inhibitory effect followed HCO_3_^−^ > H_2_PO_4_^−^ > Cl^−^ within 60 min. HCO_3_^−^ had the most obvious inhibition effect (the removal efficiency was reduced by 41.65%) because HCO_3_^−^ is a strong quenching agent of SO_4_^−^˙ and HO˙ (eqn (4) and (5));^39^ and as the reaction proceeds, the H^+^ generated by HCO_3_^−^ and SO_4_^−^˙ made the pH value of the solution decrease gradually; and decreased to 3.19 after the reaction. Moreover, a large amount of H^+^ accumulated in the cathode area of the Fe^0^–AC primary battery would be subject to competition with HCO_3_^−^, leading to the formation of H_2_O and CO_2_, which would inhibit Fe^0^–AC microelectrolysis and result in the maximum inhibition of CTC degradation. Under the inhibition of H_2_PO_4_^−^, the removal efficiency of CTC decreased by 14.2% within 60 min. The adverse effect was attributed to the complexation of H_2_PO_4_^−^ with iron,^40^ which hindered the activation of PDS by Fe^0^–AC microelectrolysis. The negative effect of Cl^−^ on CTC degradation might be attributed to the reaction between Cl^−^ and active radicals (SO^−^˙and HO˙) to form less active chlorine or hypochlorite (eqn (6)–(8)).^32^

4 HCO_3_^−^ + SO_4_^−^˙ → SO_4_^2−^ + CO_3_^−^˙ + H^+^

5 HCO_3_^−^ + HO˙ → H_2_O + CO_3_^−^˙

6 Cl^−^ + SO_4_^−^˙ → SO_4_^2−^ + Cl˙

7 Cl^−^ + Cl˙ → Cl_2_^−^˙

8 Cl^−^ + HO˙ ↔ HOCl^−^

### 3.7 Effect of humic acid on CTC degradation

**Fig. 8:**
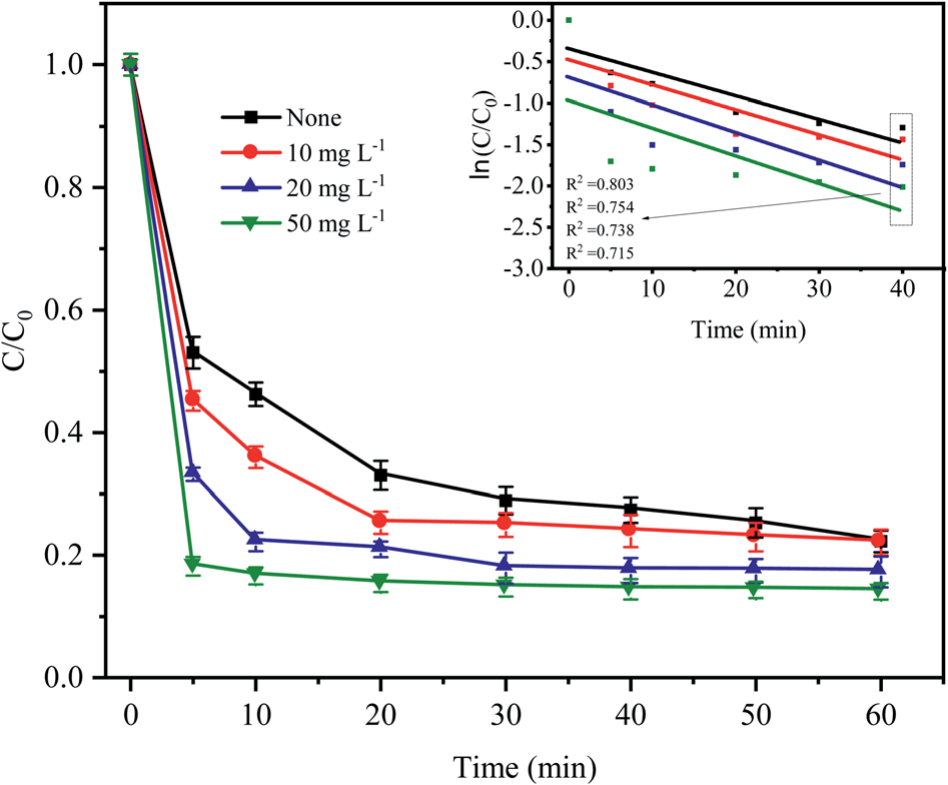
Effect of humic acid on CTC degradation in Fe^0^–AC/PDS system. Experimental conditions: [CTC] = 50 mg L^−1^, [PDS] = 2 g L^−1^, [Fe^0^] = 0.4 g L^−1^, [AC] = 0.2 g L^−1^, and pH = 5.

To simulate the existence of natural organic matter, different doses of humic acid were added into the Fe^0^–AC/PDS treatment system to investigate their effect on the degradation of CTC. As shown in Fig. 8, the degradation efficiency of CTC increased significantly when the initial concentration of HA increased from 0 to 50 mg L^−1^. Especially in the first 5 min, when the humic acid concentration was 10, 20 and 50 mg L^−1^, the CTC degradation rate increased by 7.86%, 19.88% and 34.87%, respectively, clearly showing an increasing trend. When 50 mg L^−1^ HA was added to the system, the apparent rate constant reached the maximum value of 0.0218 min^−1^, which was higher than other treatments. This is because humic acid is weakly acidic; the H^+^ on its acid group would accelerate the reaction rate of the iron–carbon primary battery, which would then activate PDS to generate more active radicals. With an increasing HA initial concentration, H^+^ increased gradually, and the reaction rate also accelerated. However, HA might act as a potential contributor to oxidant consumption or as a scavenger competing for active radicals.^32,41^ In addition, quinones and hydroxyl groups contained in HA can react with PDS, and the generated oxidized substances can also promote the degradation of CTC.^42,43^ Li *et al.* also found that the addition of HA can promote the degradation of DNT by Fe^2+^ activated PDS.^44^ In Fe^0^–AC microelectrolysis system, Fe^0^ is oxidized to form Fe^2+^ on the anode, and the synergistic activation of Fe^2+^ and HA on PDS may also be one of the reasons for promoting degradation efficiency.

### 3.8 Mineralization of CTC by the Fe^0^–AC/PDS system

**Fig. 9:**
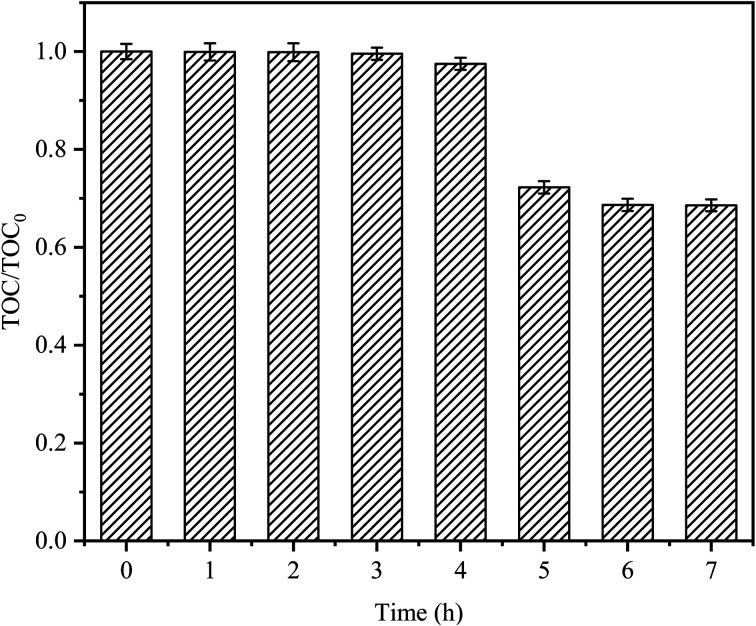
TOC removals on CTC degradation by in Fe^0^–AC/PDS system. TOC_0_ is the initial organic carbon value and TOC is the organic carbon value at *t* (h). Experimental conditions: [CTC] = 50 mg L^−1^, [PDS] = 2 g L^−1^, [Fe^0^] = 0.4 g L^−1^, [AC] = 0.2 g L^−1^, initial pH = 5, and the final pH = 3.12.

The degradation target of any organic pollutant is not only the degradation of pollutants but also mineralization. CTC mineralization was analyzed by measuring the TOC concentration from samples taken at regular time intervals. As shown in Fig. 9, there was almost no mineralization in the first 3 h; this might be because CTC would produce some intermediate compounds in the degradation process, which were difficult to mineralize.^45^ As time passed, the mineralization of CTC increased gradually, and the mineralization rate sharply increased after 4 h, with a mineralization rate of 27.75%, indicating that CTC and its intermediates could be continuously transformed into CO_2_ by the Fe^0^–AC/PDS system. After 7 h, the mineralization rate reached 31.44%. Compared with the reaction time of 5 h, the mineralization rate of CTC decreased slowly, however, there was no significant continuous decline. It might be that after a long reaction time, the Fe^0^ or PDS in the system decreased due to consumption, and the adsorption of AC on the intermediate resulted in a decrease in activity. It showed that CTC was not only oxidized and degraded into small organic compounds by the PDS activated by Fe^0^–AC microelectrolysis but also degraded into inorganic carbon forms. Jiang *et al.* obtained similar results.^39^ Compared with the degradation efficiency, it would take more time to attain a higher mineralization efficiency.

### 3.9 Identification of the activation resource

**Fig. 10:**
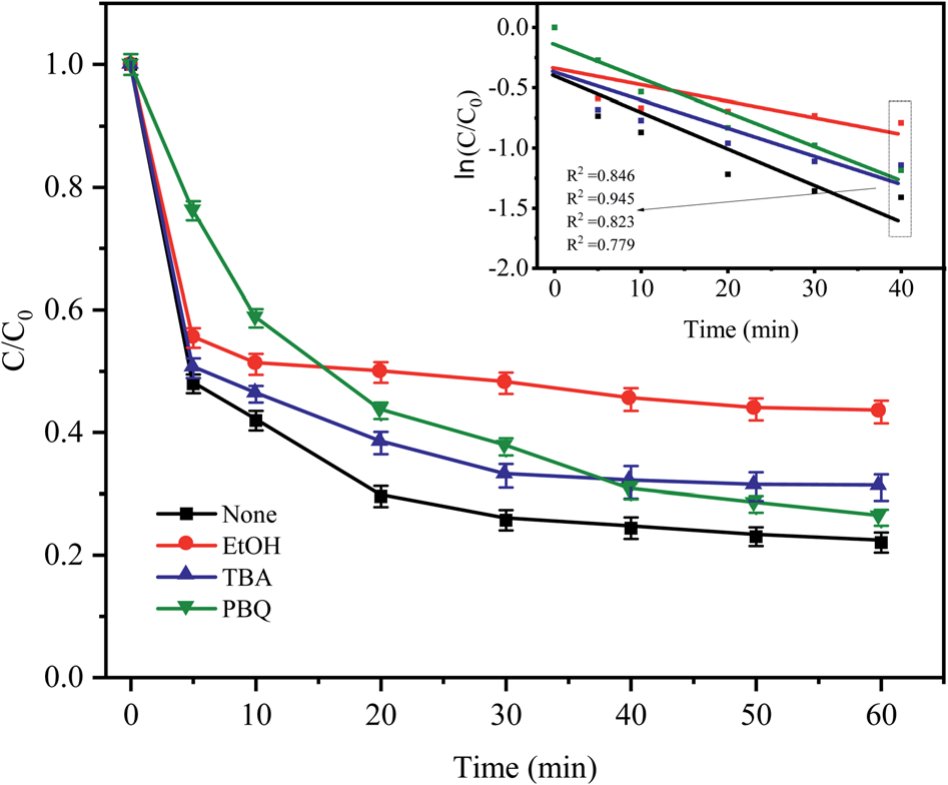
Effect of radical scavengers on CTC degradation in Fe^0^–AC/PDS system. Experimental conditions: [CTC] = 50 mg L^−1^, [PDS] = 2 g L^−1^, [Fe^0^] = 0.4 g L^−1^, [AC] = 0.2 g L^−1^, pH = 5, and [EtOH] = [*T*BA] = [*P*BQ] = 50 mM.

To identify the mechanism of CTC degradation in the Fe^0^–AC/PDS system, free radical scavengers were added to the reaction system to determine the active free radicals. EtOH containing α-hydrogen reacted with SO_4_^−^˙ and HO˙ at rate constants of 1.7 × 10^7^ and 1.2 × 10^9^ M^−1^ s^−1^, respectively. However, for *T*BA without α-hydrogen, the rate constant of SO_4_^−^˙ was 4 × 10^5^ M^−1^ s^−1^, which was 1000 times less than that for HO˙ (3.8 × 10^8^ M^−1^ s^−1^). Therefore, EtOH could effectively scavenge both SO_4_^−^˙ and HO˙, while *T*BA was a strong scavenger of HO˙.^46–48^*P*BQ is often used to inhibit O_2_^−^˙. As shown in Fig. 10, in the presence of EtOH and *T*BA, the CTC removal rate decreased by approximately 21.26% and 8.95% in comparison with the control condition. And the corresponding apparent rate constants were 0.0091 and 0.0148 min^−1^. It showed that there were both SO_4_^−^˙ and HO˙ in the system, but the contribution of SO_4_^−^˙ in the system was greater than that of HO˙. Indeed, it was shown that in most cases SO_4_^−^˙ prevails in acidic media and HO˙ predominates in alkaline media.^49^

When *P*BQ was added to the system, a CTC degradation of 23.83% was observed in the first 5 min, and the inhibition was significantly higher than that of other inhibitors. The inhibition of *P*BQ decreased gradually after 5 min of reaction, and the degradation rate of CTC as the reaction continued was 73.88%. It showed that O_2_^−^˙ was one of the main degradation species in the early stage of the reaction. Iron as the anode of a primary battery loses electrons in the microelectrolysis process. On the one hand, these electrons have sufficient reduction ability to induce O_2_ in water into O_2_^−^˙ (eqn (9)). On the other hand, PDS in solution can capture these electrons and then reduce O_2_ into O_2_^−^˙, which will attack and degrade CTC (eqn (10) and (11)). However, the inhibition effect was not obvious from the middle to the later stage of the reaction, which might be due to the consumption of O_2_ in the solution in the later stage of the reaction, resulting in the reduction of O_2_^−^˙. The results showed that O_2_^−^˙ only participated in the degradation of CTC at the beginning of the reaction, while SO_4_^−^˙ played a leading role in the degradation of CTC until the end of the reaction; however, the role of HO˙ in the whole reaction process does not seem to be important.

9 e^−^ + O_2_ → O_2_^−^˙

10 e^−^ + S_2_O_8_^2−^ → [S_2_O_8_^2−^]^−^

11 [S_2_O_8_^2−^]^−^ + O_2_ → S_2_O_8_^2−^ + O_2_^−^˙

### 3.10 Reusability of iron and carbon

**Fig. 11:**
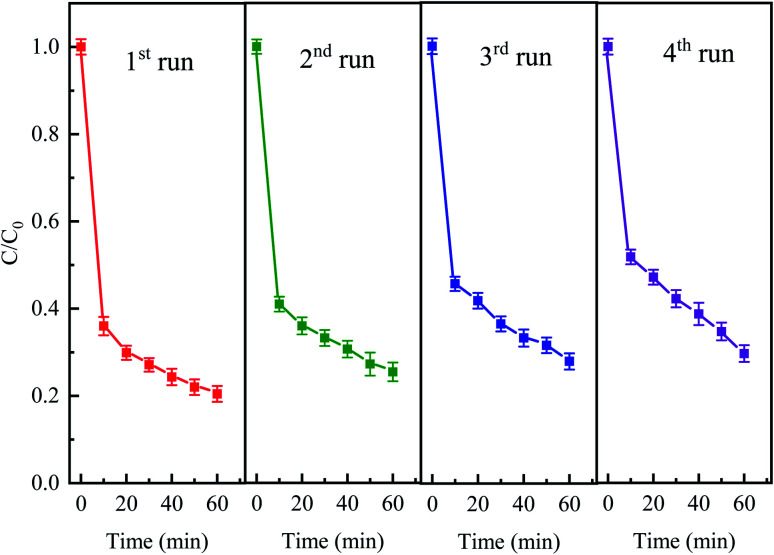
The degradation of CTC in Fe^0^–AC/PDS system within the successive four cycles. Experimental conditions: [CTC] = 50 mg L^−1^, [PDS] = 2 g L^−1^, [Fe^0^] = 0.4 g L^−1^, [AC] = 0.2 g L^−1^, and pH = 5.

The stability and reusability of the catalyst is an important factor to be evaluated. The stability experiments were conducted for four successive cycles under the following conditions: 50 mg L^−1^ CTC, 0.4 g L^−1^ Fe^0^, 2 g L^−1^ PDS and pH 5 for a reaction period of 60 min. After every run, the iron and activated carbon were filtered and separated with magnets and then washed with deionized water. As shown in Fig. 11, the degradation efficiencies of CTC for every recycle slightly decreased. The elimination efficiency of each repeated degradation experiment was 79.56%, 74.57%, 72.07% and 70.35%, respectively. The results showed that CTC degradation efficiency was still over 70% after fourth cycle in Fe^0^–AC/PDS system. The decrease in Fe^0^–AC/PDS system efficiency can be attributed to following reasons: (1) Fe^2+^ was leached in a large amount during each cycle and the active sites on the catalyst surface were decreased; (2) adsorption of CTC on the surface of iron and carbon which prevented the contact of the catalyst and PDS.^50^ The results demonstrated the excellent stability and retrievability of the iron and carbon materials in the Fe^0^–AC/PDS system, which was beneficial for the reusability of the catalyst.

### 3.11 Possible mechanism analysis

Fe^0^–AC/PDS process was a complex reaction system. Under single factor conditions, Fe^0^, AC, PDS and Fe^0^–AC have no obvious effect on CTC removal; but in the Fe^0^/PDS system, the CTC degradation effect was significantly increased. It is worth noting that when AC was added to the Fe^0^/PDS system, the CTC degradation effect was the best. According to the outcomes in the literature, electrolysis has a positive activation effect on PDS. In the presence of an electric field, pollutants gathered on the surface of the anode and cathode and were more conducive to undergoing an oxidation reaction.^22^ This was because the Fe^0^ and AC in the solution formed a primary battery, which would activate the electron transfer in the solution through microelectrolysis, accelerate the dissolution of anode Fe^2+^ and promote the formation of a new cathode ecology [H]. With the microelectrolysis process, PDS could be well activated and generate a high redox potential for SO_4_^−^˙ and O_2_^−^˙, which made CTC degradation fast and efficient.

The possible mechanism of CTC degradation in the Fe^0^–AC microelectrolysis-activated PDS system includes the following: (1) in the anode of the Fe^0^–AC primary battery, the electron lost from Fe^0^ can reduce O_2_ in water to generate O_2_^−^˙, which then attacks CTC to complete degradation; (2) the anode Fe^0^ dissolves Fe^2+^ through the microelectrolysis of the primary battery, and Fe^2+^ activates PDS to generate highly active SO_4_^−^˙, which leads to CTC degradation; (3) the active sites on the AC surface can activate PDS to produce SO_4_^−^˙; and (4) H^+^ can obtain electrons in the cathode of the Fe^0^–AC primary battery and form a new ecology with strong chemical activity [H], which can react with CTC.

## 4. Conclusions

The effect of PDS activation by Fe^0^–AC microelectrolysis on CTC degradation was investigated. The treatment efficiency of CTC in different systems was as follows: Fe^0^–AC/PDS > Fe^0^/PDS > AC/PDS > Fe^0^–AC > AC > Fe^0^ > PDS. The best Fe^0^ dose in the Fe^0^–AC/PDS system was 0.4 g L^−1^, and the degradation rate of CTC increased with an increasing PDS concentration. The pH of the solution had a significant influence on the degradation ability of the system, and acidic conditions were the most favorable for the reaction. The system could degrade CTC in a range of 20 to 150 mg L^−1^ with a degradation rate of more than 70%. The presence of Cl^−^, HCO_3_^−^, H_2_PO_4_^−^ could inhibit the degradation of CTC in the system, in which HCO_3_^−^ had the most obvious inhibition. Humic acid could promote the degradation of CTC. The mineralization rate of CTC was 31.44% within 7 h, which showed that CTC was not only oxidized into small organic compounds but also degraded into inorganic carbon. Free radical identification experiments demonstrated that O_2_^−^˙ only played a leading role in the early stage of the reaction, while SO_4_^−^˙ played an important role in the whole reaction process. Iron and carbon materials had good stability and recoverability in the Fe^0^–AC/PDS system, which was conducive to catalyst reusability.

## Conflicts of interest

There are no conflicts to declare.

## Supplementary Material
